# Dr. Pancoast’s Case of Excision of the Elbow-Joint

**Published:** 1842-09-24

**Authors:** J. Pancoast

**Affiliations:** Professor of Anatomy in Jefferson Medical College, &c.; Philadelphia


MEDICAL EXAMINER.

NEW SERIES.

No. 39.] PHILADELPHIA, SEPTEMBER 24, 1842. [Vol. I.

*A Case of Excision of the Elbow-Joint.* By J. Pancoast, M. D., Professor of Anatomy in Jefferson Medical College, &c.

Augustus Heyl, of Mount Holly, N. J., æt. 32, was thrown from his wagon in the summer of 1841, and received the force of the fall upon his right arm. A dislocation of the elbow-joint, backwards and upwards, was the consequence, attended, as he believes, with fracture of one of the bones. Reduction was made of the bone without difficulty, by an intelligent physician in the neighbourhood, and the patient in a few days resumed his occupation of distributing bread about the country, without any other protection to his arm than a sling. As the result of the imprudence, intense swelling and inflammation set up in the joint, followed by abscess, which discharged externally through several sinuses on the back part of the joint. Through these openings a discharge of matter was nearly constant, and a probe passed in traversed the cavity of the articulation, and grated against the carious extremities of the ulna and humerus.

From time to time, as the fistulous orifices closed, successive attacks of inflammation came on, ending in the formation of a fresh abscess, attended with great constitutional irritation, and in two instances with emprosthotonos. There was great swelling and thickening of the whole elbow, and the integuments at the posterior part of the joint were of a violet colour. The arm above shrunk to about half the size of its fellow of the opposite side. The joint became rigid with the forearm in the extended position. The radius was forcibly pronated and immoveable. To relieve the agony which he constantly suffered, the patient was in the habit of taking large quantities of laudanum. He became nervous and irritable; and though successively under the charge of various practitioners, here and elsewhere, he found little relief, as he was disinclined to follow out any course of treatment proposed. Finding his constitution giving way, he came to the city for the purpose of having his arm amputated, and placed himself under my care.

From the age of the patient, (his constitution being naturally good,) I determined to try to save the limb, which was the right, by excision of the elbow-joint; by which means I hoped at all events to get rid of the diseased bone, to be able to place his arm in the flexed position, so that it might be more useful to raise the forearm from its prone position, and possibly re-establish motion at the joint.

His journey to town, and subsequent walking about with his arm on a pillow, led to a new attack of inflammation, with great constitutional disturbance. The swelling, which was immense around the joint, extended up two-thirds the length of the arm, and was attended with a severe attack of pneumonia, which endangered his life. By appropriate treatment, and freely opening the abscess of the joint, he recovered. By June 14th his general condition had so far improved, that I proceeded to the operation for excision, assisted by Drs. Peace, Davis, Bibighaus and others. The patient was laid on a bed with his face downwards; a tourniquet was applied on the arm, which was extended over the side of the bed, and held by an assistant. Another assistant supported the shoulder, and restrained the movements of the patient. The joint was exposed after the method of Moreau and Syme. I entered the bistoury perpendicularly, with its back nearly in contact with the ulnar nerve, and laid the joint widely open with a cross just above the end of the olecranon. Two longitudinal incisions were extended above, and two below from the ends of the cross cut, each an inch and a half in length, so that they formed the shape of the letter H. The flaps were dissected up and reflected. The periosteum was so loosened by suppuration, over the end of the humerus, that it stripped up without difficulty; but such an effusion of osseous matter had taken place in the substance of the flap, that the reflection was difficult; if let slip, it fell back with an elastic rebound. I next removed the olecranon with the amputation saw. The articular surfaces of the humerus and ulna were now found to be carious and softened; the synovial membrane of the joint, and the loose tissue of the greater sigmoid cavity in a state of pulpy fungus. To remove the articular surface of the humerus (the bone above this, though inflamed and softened, not appearing carious,) I applied Barton's saw just at the margin of the epicondyles, and divided the bone nearly through with a strong pair of cutting forceps; partly by splitting, and partly by cutting, the section was completed. The loosened end was seized with a pair of large tooth forceps, and removed with a few strokes of the knife. The head of the ulna, which was very soft, and filled with fatty matter, was removed in the same manner. The whole base of the coronoid process it was not necessary to take away. Its top, and its whole articular face, which was carious, was divided with the cutting bone forceps, and twisted off from the tendon of the brachialis anticus with the large tooth forceps. This was the most painful part of the operation. The head of the radius, on which the cartilage was softened, was cut off in like manner and removed. All the pulpy synovial and cellular tissue was carefully dissected away. With some effort at first the forearm could now readily be flexed. The thumb was placed in the middle position, between pronation and supination; but to enable me to do this, it was necessary first to divide with the knife the orbicular ligament of the radius. All the carious bone being now removed, the cavity was sponged clear of blood. Two small arteries were tied, and the flaps brought together with six sutures—two in the transverse, and four in the longitudinal incisions. The elbow was placed on a pillow slightly bent, so as to avoid straining the parts at the transverse incision; simple dressings were applied, and a patent elbow splint, well padded, and secured with a figure of 8 bandage, placed on the back part of the joint, so as to prevent all motion. The arm was secured to the pillow with a second roller, and laid on an inclined plane made with pillows. Eight or ten ounces of blood were lost during the operation. The patient was directed to take a quarter of a grain of morphia in an ounce of camphor water every three hours, and to keep the dressings of the elbow well soaked with a strong solution of lead water and laudanum.

June 15. The patient has passed an excellent night; better than he has done for many months. The arm perfectly free from pain. At my evening visit the same day, I found him with the symptoms of incipient pneumonia. Pain in the breast; frequent cough; subcrepitant rhonchus. Directed a table-spoonful of the following mixture every hour and a half. ℞. Infus. Polyg. Seneg. ℥viij.; Oxy. Scill. ℥j.; Tr. Lob. Infl. ℥j.; and a mustard plaster to the chest.

The lotion to be continued to the arm as before, from which there had been a slight oozing of blood.

16. Has been slightly nauseated; expectorates freely; some fever; no pain in the arm; subcrepitant rhonchus rather less diminished; some pain still in the chest. Directed a purgative enema; a blister to the front of the chest.

Expectorant mixture to be continued.

17. Condition much improved. Blister has drawn well; says it gave him much more pain than the operation on the joint. Chest free from pain. No unpleasant feeling in the elbow-joint; slept well; expectorates freely; pulse soft and good. Weak chicken water for diet. Mixture to be continued at lengthened intervals, with a quarter of a grain of Sulph. Morphiæ at bed time.

18. Condition excellent. Cough almost entirely gone; some expectoration still; slept well; has a good appetite, and no fever or pain in the arm. To-day dressed the wound. On removing the bandage it was found no suppuration had taken place, nor was there the slightest redness or swelling in the flaps. At all the points of junction of the flaps, (which were not brought in consequence of their rigidity into apposition at the time of the operation, the free space at the transverse wound being full three-sixteenths of an inch in width,) some coagulated blood was found interposed level with the skin, and solid and firm in its character. This I carefully refrained from disturbing. The simple dressing and splint was applied as before, and the same local treatment of strong lead water and laudanum directed.

The expectorant mixture to be continued.

19. Condition excellent in every respect; says his arm has been less painful since the operation, than it has ever been since the reception of the injury. Patient is up and moving about the room, with his arm supported on a pillow.

Expect. mixture discontinued.

20. The day being mild and fair, the patient felt so well as to walk out, and met me, at the time of my visit, a square and a half from his residence. To-day dressed the arm again. There was still no suppuration, except at the points of the sutures; nor the least change of colour in the flaps. The outer black surface of the blood, which filled up the interstices of the linear wounds, had peeled off here and there in a thin coat, leaving below it a mass of grayish red fibrine, which was unquestionably the effused and undisturbed blood adherent to the margin of the flaps, and in the first state of organisation. The parts were merely wiped dry with a piece of soft lint, and the sutures divided with a pair of scissors, and the same dressing and treatment as before reapplied.

21. Patient has obstinately continued to walk about; has suffered no pain whatever in his arm, as the joint has as yet been kept in a state of perfect immobility. On removing the dressings a slight degree of suppuration was obvious along the track of the wounds, and much more abundant at the points of the sutures. The loosened sutures were now withdrawn. The parts sponged lightly with warm water, and the dressings reapplied the same in every respect as before. During the last three dressings the forearm had been at each time a little more flexed, so that it was now in a middle position between that of extension and a right angle.

Simple dressings were applied.

23. To-day the two ligatures of the vessels were taken away; at the point at which they were withdrawn there was some purulent discharge. At every other part of the wound the union was nearly complete, and may be considered as having taken place by first intention. The limb was now brought nearly to a right angle. The patient was exhibited to-day at the Clinique of the Jefferson Medical College. He left against my wishes for his home, and was not seen by me again for a week; he was directed daily to make passive motion of the arm.

July 1. A fistulous orifice of small size exists at the place from which the ligatures were withdrawn. At every other part the union remains perfect, and the shape of the elbow presents very much the natural appeararance. The movement of the joint, which has been always attended by more or less pain, has been imperfectly kept up; flexion and extension, pronation and supination, can, however, be made to a considerable extent; and there is no question, if daily made under the care of a surgeon, that a most useful joint may be formed. The patient was enjoined to keep up the motion. But he is so well satisfied with his limb in its present condition, as it is bent at a right angle, free from pain and useful, and his health good, that there is reason to fear he will not subject himself to further pain to improve the motion of the joint.

8. The patient again came to the city. The small fistulous orifice had closed. The thumb retained its upright position, though the movements of pronation and supination not having been properly kept up, were less full than on the last visit. The forearm, by a little effort, can be flexed and extended to nearly the usual extent. From this period I have not seen or heard of the patient; and the movements of the elbow, which he now possesses, must be of a kind proportioned to his perseverance in keeping up the passive and voluntary motion of the joint, which were enjoined upon him as strongly as possible, inasmuch as he seemed indifferent to the result.

### *Remarks.*

In this case I departed from the usual method of after treatment. No poultices or warm applications were at any time made about the joint. The plan of Mr. Syme, to open the wound on the second day after the operation, to sponge out the clotted blood, and of course provoke abundant suppuration, I most carefully abstained from, as I was desirous of producing the union of so extensive a wound, in a constitution which had been so much impaired, as far as possible by first intention. By the perfect rest of the part, the length of the interval between the operation and the second dressing, and especially by the use of the lead water and laudanum mixture, this desired result was attained to a greater extent than I am aware has ever attended an operation of the sort. The composition of the mixture I employ is, Ex. Saturni, ʒss.; Tr. Thebaicæ, ℥ij. to a pint and a half of water. I have found this so useful after great operations in checking pain, preventing inflammation, and disposing to union by first intention, and, as I am well assured, occasionally to the organisation of the fibrine of layers of effused blood, that I feel desirous of attracting to the practice the notice of the profession. Under its influence, I have had in one case the flap from the forehead, in rhinoplasty, to unite completely, by first intention, to the margins of the nostril.

In the present case the articular heads of the bones only were removed, and there was, therefore, necessarily, far less danger of having the vacillating unsteady fibro-ligamentous joint apt to follow where a large portion of the bones were removed, than a somewhat rigid but rectangular joint—a lesser evil of the two. In many cases reported, I am disposed to believe an unnecessary amount of bone has been removed; and, at least, by practising the operation at an earlier period than has been usually done, all the diseased synovial tissue, cartilage, and articular faces of the bones might be removed without the necessity, as in the above case, of going on the one hand above the epicondyles, or on the other, so low as to take away the place of insertion of the brachialis anticus on the root of the coronoid process and the ulna adjoining it; leaving, in consequence, a much better opportunity for the preservation of a useful limb.

*Philadelphia*, Sept. 15, 1842.

